# Evaluating the Inhibitory Effects of Probiotic Bacteria and Propolis Extracts on the Growth and Histopathological Changes in Gastric Tissues of *Helicobacter pylori* Challenged Wistar Rats

**DOI:** 10.5812/ijpr-148158

**Published:** 2024-12-25

**Authors:** Roghayeh Kiani, Naheed Mojgani, Farzad Kobarfard, Parvaneh Saffarian, Seyed Abdulmajid Ayatollahi, Mona Khoramjouy

**Affiliations:** 1 Department of Biology, Science and Research Branch, Islamic Azad University, Tehran, Iran; 2 Biotechnology Department, Razi Vaccine and Serum Research Institute, Agricultural Research, Education and Extension Organization, Karaj, Iran; 3 Department of Medicinal Chemistry, School of Pharmacy, Shahid Beheshti University of Medical Sciences, Tehran, Iran; 4 Phytochemistry Research Center, Shahid Beheshti University of Medical Sciences, Tehran, Iran

**Keywords:** *Helicobacter pylori*, Probiotics, Propolis, Antibacterial Activity, Histopathological Studies, Gastric Tissues, Wistar Rats

## Abstract

**Background:**

* Helicobacter pylori* is a significant contributor to a range of gastrointestinal conditions, with conventional treatment methods primarily relying on antibiotics. However, the rise of antibiotic-resistant strains has necessitated the exploration of alternative therapeutic approaches.

**Objectives:**

To determine the *in vitro* antibacterial potential of probiotic bacteria (*Lacticaseibacillus rhamnosus* BLRH 260 and *Limosilactobacillus reuteri*) and four propolis extracts against *H. pylori* and to analyze their impacts on body weight index and histopathological changes in *H. pylori*-challenged Wistar rats.

**Methods:**

The inhibitory effects of probiotic bacteria (*L. rhamnosus* BLRH 260 and *L. reuteri*) and propolis extracts on the growth of *H. pylori* were evaluated using an agar well diffusion assay. In vivo analysis involved fifty-four male Wistar rats (200 - 250 g) infected with an *H. pylori* suspension (10^8^ CFU/mL) and orally administered propolis or probiotics (10^8^ CFU/mL) via gavage for 21 days. The effects of different treatments on body weight and histopathological changes in gastric tissue samples were assessed, and the results were statistically analyzed.

**Results:**

The tested propolis extracts and the supernatant fluids from the mentioned probiotic strains showed significant antibacterial activity against *H. pylori* in the agar well diffusion assay, with notable variations. In vivo, the findings demonstrated that oral administrations of propolis and probiotics, either separately or in combination, led to significant increases in body weight and amelioration of histopathological changes in gastric tissue samples, particularly in terms of erosion depth, hemorrhagic inflammation, and apoptosis in the infected animals. Histopathological differences between antibiotic-treated animals and those receiving other treatments were observed, with significant differences.

**Conclusions:**

The results of this study underscore the potential therapeutic benefits of propolis and probiotics in addressing *H. pylori*-induced gastropathy. Additional research is necessary to clarify the mechanisms involved and to refine dosage and treatment protocols for optimal effectiveness.

## 1. Background

*Helicobacter pylori *is a spiral-shaped bacterium that colonizes the gastric mucosa of more than half of the world's population (1). This bacterium has been identified as a major causative agent of various gastrointestinal disorders, ranging from mild symptoms like superficial gastritis to more severe conditions, including chronic gastritis, peptic ulcers, and gastric cancer. The treatment strategies for *H. pylori* infections typically involve antibiotics, but the emergence of antibiotic-resistant strains has prompted a search for alternative therapeutic options (2, 3). Several natural components, such as plant extracts, essential oils, phages, probiotics, and bee products, have been shown to possess activity against *H. pylori*, provide gastroprotective benefits, and demonstrate anti-inflammatory properties (4, 5).

Probiotic bacteria have gained significant attention due to their safety and their ability to restore and maintain a healthy gut microbiota (6). Probiotics are defined as live biotherapeutic agents, including bacteria and yeasts, that confer health benefits when consumed in adequate amounts (FAO/WHO, 2002). These live microorganisms, primarily belonging to the *Lactobacillus* and *Bifidobacterium* genera, offer a range of health benefits by modulating the gut environment, improving mucosal barrier function, and regulating immune responses. A significant property of probiotic bacteria is their antimicrobial activity against various pathogens, including *H. pylori*, making them potential candidates for alternative treatment approaches (7). 

It has been scientifically proven that probiotics could control *H. pylori* infections, as some *Lactobacillus* strains are able to survive in the acidic conditions of the human stomach for extended periods of time. The antibacterial effects exerted by these strains could be related to their ability to produce antimicrobial substances such as bacteriocins or other metabolic byproducts, such as lactic acid and acetic acid, which contribute to a lower pH that could have detrimental consequences on the growth of pH-sensitive pathogens. Additionally, some *Lactobacillus* species might compete with *H. pylori* for attachment to epithelial cells (4). Numerous clinical and fundamental studies have indicated that specific strains of probiotics can eradicate *H. pylori* while significantly mitigating side effects associated with treatment, thereby enhancing patient adherence to the therapeutic regimen. These attributes have led to the recognition of probiotics as a potentially valuable adjunctive therapy to combat *H. pylori* infection (8).

Aside from probiotics, propolis is another natural compound that has been recognized as a natural remedy for a range of infectious diseases, including those caused by *H. pylori* (4, 9). Propolis, a resinous substance collected by honeybees from plant sources, possesses a diverse array of bioactive compounds (10). These compounds include phenolic acids, flavonoids, terpenes, and other polyphenols, which contribute to its antimicrobial, anti-inflammatory, and antioxidant properties. Studies have suggested that propolis extracts exhibit inhibitory effects against *H. pylori*, making them potential natural alternatives to combat this infection, which is associated with gastrointestinal disorders (11, 12). Propolis generally exhibits bacteriostatic properties against various bacterial genera; however, it may demonstrate bactericidal effects at elevated concentrations (13).

Although previous studies have investigated the inhibitory effects of probiotics and propolis extracts on *H. pylori* in in vitro models, there remains limited research data regarding their efficacy in in vivo models (12). Nonetheless, to our knowledge, this study for the first time describes and evaluates the synergistic actions of probiotics alongside various concentrations of propolis in animal models challenged with *H. pylori*. Animal models, particularly those employing Wistar rats, provide valuable insights into the complex interactions between the host, pathogen, and potential therapeutic agents (14, 15). 

## 2. Objectives

The present study aimed to evaluate the inhibitory effects of probiotic bacteria and propolis extracts on the growth of *H. pylori*. By administering specific strains of probiotic bacteria and propolis extracts orally to the experimental rats, their impact on the colonization of *H. pylori* in the gastric mucosa was analyzed.

## 3. Methods

### 3.1. Bacterial Strains and Culture Conditions

*Lacticaseibacillus rhamnosus* BLRH 260 and *Limosilactobacillus reuteri* BLRE 269 were kindly provided by a local probiotic manufacturer, Biorun Co., Karaj, Iran. The two strains were propagated in MRS (DeMan Rogosa and Sharpe, Merck, Germany) broth media under anaerobic conditions for 24 hours at 37ºC. *Helicobacter pylori* ATCC 43504 was obtained from Pasture Institute, Tehran Iran. *Helicobacter pylori* clinical isolates were cultured on *Brucella* agar (BA, Oxoid, USA) medium with 7% v/v horse blood, under microaerobic conditions (GasPak, Oxoid) at 37°C for 5 - 7 days (15). For long-term storage, stock cultures were maintained at -70ºC in 20% glycerol.

### 3.2. Propolis Collection and Preparations of Ethanolic Extracts

Propolis samples were procured from beekeepers in four different regions of Iran: P1 (Sangestan-Hamadan region), P2 (Khorramabad area), P3 (Karaj), and P4 (Gari-Nahavand mountain range), during December and January of 2020. All samples were collected in plastic bags wrapped in foil and kept at -20°C for further analysis. To prepare ethanolic extracts, the frozen propolis samples were ground into powder using a mortar and stored at -80°C until further analysis. Twenty grams of propolis powder were added to 500 mL of 70% ethanol, and the samples were kept in the dark overnight at room temperature. The samples were then filtered using Whatman filter paper No. 4. This step was repeated three times to achieve a suspension without residues. After extraction, the samples were stored in the dark at -20°C until further analysis. The percentage yield of the propolis was calculated using the following formula:

Percentage yield (%) = (Amount of pure product recovered/Amount of crude material used) × 100

The suspensions were filtered and concentrated in a rotary evaporator under reduced pressure. The prepared extracts in glass tubes were covered with foil and refrigerated for further use. For experiments, the four extracts were mixed in equal proportions and used in the treatment groups (16).

### 3.3. Screening for Antimicrobial Activity

The antibacterial activity of propolis and the mentioned probiotic strains against *H. pylori* was determined by disc diffusion and agar well diffusion assays as described earlier (17). 

#### 3.3.1. Preparation of Cell-Free Supernatant Fluids of Probiotic Bacteria 

Cell-free supernatant (CFS) from *L. rhamnosus* and *L. reuteri* was prepared for antibacterial screening. Freshly grown cultures of the respective probiotic bacteria were centrifuged at 6000 x g for 20 minutes at 4ºC. The collected supernatant fluids were filter sterilized using a 0.2 µm filter membrane (Sartorius AG, Göttingen, Germany) and used for further analysis (16).

#### 3.3.2. Agar Well Diffusion Assay

For the agar well diffusion assay, wells with 5 mm diameters were punched into the agar plates overlaid with indicator cultures. The indicator cultures were prepared by adjusting the overnight-grown cultures to 1.8 × 10^8^ log CFU/mL (2 McFarland standard). To each well, 100 microliters of the CFS and the respective propolis extracts were added, and the plates were temporarily incubated at refrigerated temperatures (approximately 2 to 4 hours at 4ºC) to allow maximum absorption of the extracts. The plates were then incubated under microaerophilic conditions (5% O_2_, 10% CO_2_, and 85% N_2_) at 37°C for 5 to 7 days. Antimicrobial activity was evaluated by measuring the zone of inhibition around the wells. Each test was performed in triplicate. Ethanol (70%) was used as the control solvent.

### 3.4. In Vivo Animal Study

#### 3.4.1. Ethics

Animal studies were performed under the observation of the Committee on Animal Experiments of Shahid Beheshti University of Medical Sciences, with approval code IR.SBMU.RETECH.REC.1401.321. All animal experiments complied with the ARRIVE guidelines and were carried out in accordance with the EU Directive 2010/63/EU for animal experiments.

#### 3.4.2. Animals and Diets 

Fifty-four male Wistar rats (200 - 250 g), purchased from the Pasteur Institute (Tehran, Iran), were housed in a temperature-controlled environment at 22 ± 2°C with 50 ± 5% humidity and a 12-hour light/dark cycle. Prior to the initiation of the experiment, the animals were given one week to acclimate to their new environment (18).

#### 3.4.3. Control and Treatment Groups

The rats were randomly assigned to nine treatment groups, consisting of six rats each. *Helicobacter pylori *infection was induced in the rats by administering 1 mL of *H. pylori* suspension (10^8^ CFU/mL) twice daily for three days via intragastric tubes (gavage). Probiotic suspensions of *L. rhamnosus* and *L. reuteri* were prepared at concentrations of 10^8^ CFU/mL (18).

The control and treatment groups were as follows: (1) Control group (healthy animals) orally received an equivalent volume of PBS; (2) HP group (*H. pylori*-infected animals); (3) HP + Pa group [*H. pylori* + Propolis (75 mg/kg)]; (4) HP + Pb group [*H. pylori* + Propolis (150 mg/kg)]; (5) HP + Pc group [*H. pylori* + Propolis (300 mg/kg)]; (6) HP + Pro group [*H. pylori* + mixed probiotic strains (10^9^ CFU/mL)]; (7) HP + Pb + Pro group (*H. pylori* + Propolis [150 mg/kg) and mixed probiotics (10^9^ CFU/mL)]; (8) HP + Pc + Pro group [*H. pylori* + Propolis (300 mg/kg) and mixed probiotics (10^9^ CFU/mL)]; (9) *H. pylori* + omeprazole (20 mg/kg) + amoxicillin (50 mg/kg) + clarithromycin (25 mg/kg) group (positive control). The challenged group animals received the mentioned concentrations of propolis and/or probiotics for 21 days via gavage.

#### 3.4.4. Animal Body Weight 

The weight of the animals in all treatment groups was assessed on days 1, 7, 14, and 21 of the experimental period.

#### 3.4.5. Histopathology

Rats were sacrificed at the end of the experiment, and gastric tissues were collected. The gastric tissues were preserved in a 10% formaldehyde solution. Sections were cut at a thickness of 5 μm after being embedded in paraffin. Hematoxylin-eosin (HE) and Giemsa staining techniques were then used. Histopathological findings in HE staining, including erosion depth, hemorrhage, inflammation, and apoptosis, were recorded and scored as follows: Erosion depth of up to 1/3: Score 1, erosion depth of up to 2/3: Score 2, Erosion depth of total: Score 3, focal hemorrhage: Score 1, mild hemorrhage: Score 2, Severe hemorrhage: Score 3, light inflammation: Score 1, mild inflammation: Score 2, severe inflammation: Score 3, light apoptosis: Score 1, mild apoptosis: Score 2, severe apoptosis: Score 3 (18, 19). Subsequently, three different researchers measured all pathological index cases to increase objectivity.

### 3.5. Statistical Analysis

GraphPad Prism software was used to analyze the data. The mean ± standard error of the mean (SEM) for all obtained results was presented. One-way and two-way analysis of variance (ANOVA) were used to compare all groups, followed by Bonferroni’s test. Differences were considered significant at P < 0.05.

## 4. Results

### 4.1. In Vitro Antibacterial Activity

As depicted in Table 1, the tested propolis extracts and supernatant fluids from the mentioned probiotic strains demonstrated significant antibacterial activity against *H. pylori* during the agar well diffusion assay. Among the tested propolis ethanolic extracts, P4, obtained from the Gari Nahavand mountain range, showed the highest antibacterial activity against the pathogen, followed by P1, collected from the Sangestan Hamedan region, which exhibited a zone diameter of approximately 14 mm (P ≤ 0.05). Compared to P1 and P4, the antibacterial activity demonstrated by P2 and P3 was lower and insignificantly different (P > 0.05). A slight increase in antibacterial activity was observed in mixed propolis samples, but the activity was insignificantly different from P4 (P > 0.05).

**Table 1.** Antimicrobial Activity of Propolis Ethanolic Extracts and Probiotic Bacteria (*Lacticaseibacillus rhamnosus *and *LimosiLactobacillus reuteri*) Against *Helicobacter pylori *by Agar Well Diffusion Assay ^a, b, c, d^

Variables	Values
**P1 **	15.05 ± 0.15 ^B^
**P2**	12.95 ± 0.18 ^C^
**P3**	12.22 ± 0.25 ^C^
**P4**	16.85 ± 1.48 ^A^
**P**_**mix**_	17.03 ± 2.33 ^A^
***Lacticaseibacillus** **rhamnosus***	15.36 ± 0.31 ^B^
***LimosiLactobacillus** **reuteri***	10.95 ± 1.20 ^D^
**L**_**mix**_	17.87 ± 1.89 ^A^

^a^ Inhibitory zone diameters were measured in millimeters (mm).

^b^ P: Propolis samples (P1: Sangestan-Hamadan region, P2: Khorramabad area, P3: Karaj and P4: Gari-Nahavand mountain range, P_mix_: Mixtue of all 4 propolis extracts), L_mix_: Mixed supernatant fluid of the two probiotic strains.

^c^ Results are indicative of three independent experiments (triplicates).

^d^ Means indicated with superscript capital letters shows significance of the results statistically analyzed (P < 0.05).

Among the tested probiotic strains, the supernatant fluid from *L. rhamnosus* showed higher antibacterial activity compared to that of *L. reuteri*, and the differences were highly significant (P < 0.05). After mixing the supernatant fluids from the two probiotic strains, the activity was significantly enhanced and became almost comparable to the activity of the propolis mix. Compared to the other tested compounds, *L. reuteri* showed the least antibacterial activity against the pathogen, with significant differences (P < 0.05).

### 4.2. In Vivo Assay in Wistar Rats

#### 4.2.1. Body Weight

**Figure 1.**
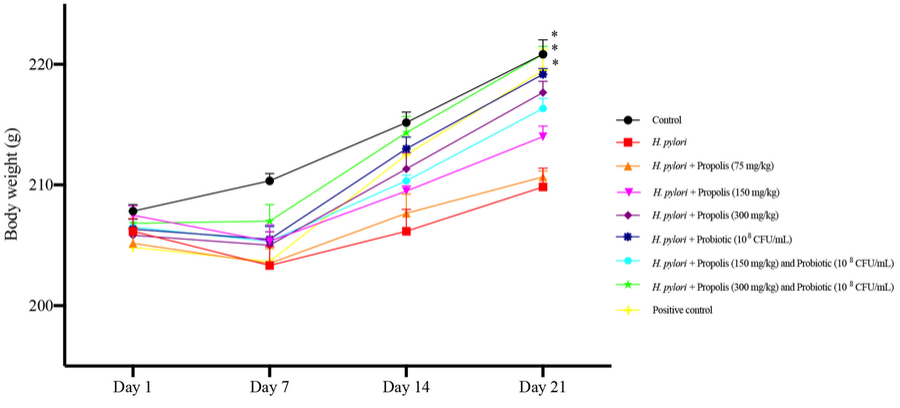
Effects on body weight of Wistar rats fed different concentrations of propolis extracts (75, 150 and 300 mg/kg) and the Probiotics (10^8^ CFU/mL), either alone or in combination for 21 days. Data were analyzed at 1, 7, 14 and 21 days of experiment. The results were analyzed by two-way ANOVA followed by Bonferroni’s test. Data are described as mean + SEM (n = 6). *P < 0.05 determined by comparing to the *Helicobacter pylori *group.

The body weight of the animals in different treatment groups is shown in Figure 1. As evident, the animals receiving the highest concentrations of propolis (300 mg/kg) and the mixed probiotics showed increased body weight gain compared to other treatment groups after 21 days (P < 0.05). An effect of time and treatment on body weight was observed, and this interaction was significant [F (3.692, 18.46) = 4.475]. During the initial challenge period (0 to 7 days), a decline in body weight was recorded for all animals, with the highest loss observed in *H. pylori*-infected animals and the lowest in control group animals receiving only PBS (P < 0.05). However, after 7 days of treatment, a gradual increase in weight gain was seen in all animals, which was more significant in those receiving the highest concentrations of propolis (300 mg/kg) and the probiotics. During the recovery phase, an increase in the rats' appetite was observed, leading to substantial weight gain in these subjects. The notable variation on day 21 indicates that the combination of propolis and probiotic treatment positively influences body weight management. Overall, in animals receiving either propolis or probiotics, the increase in weight was more pronounced in animals receiving probiotics compared to propolis (P < 0.05).

#### 4.2.2. Histopathological Studies

**Figure 2.**
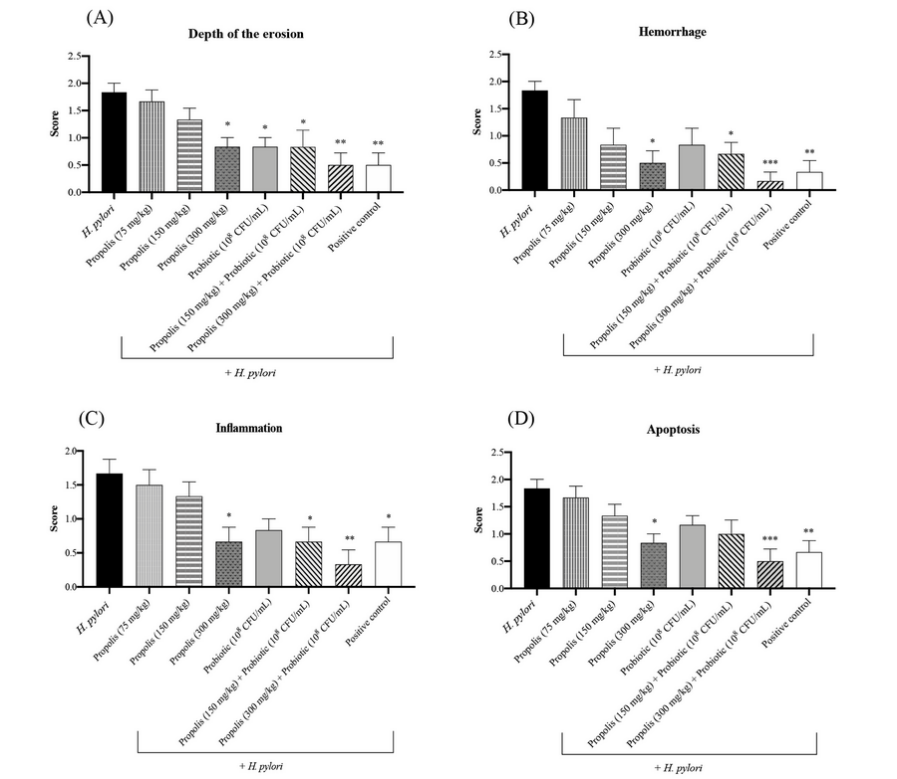
A, Histopathological alterations in gastric tissues including the depth of erosion; B, hemorrhage, C, inflammation; and D, apoptosis in animals in different treatment groups. Positive control group (treated with amoxicillin 50 mg/kg + clarithromycin 25 mg/kg + omeprazole 20 mg/kg). *P < 0.05, **P < 0.01 and ***P < 0.001 compared with the control group. One-way ANOVA was used to evaluate the data, and then Tukey's post-hoc test was performed. Data is presented as mean ± SEM (n = 6).

Gastric tissues collected from the animals in different treatment groups were studied for any histopathological changes at the end of the experimental period. Considering the microscopic scores using H&E and Giemsa staining in the *H. pylori* challenged rats, the results for the depth of erosion, hemorrhage, inflammation, and apoptosis assessment showed that the control group received a score of 0 (healthy animals receiving only PBS). As shown in Figure 2, the depth of erosion in different treatment groups was statistically significant (P < 0.01, P < 0.01, P < 0.05, and P < 0.01). Compared to the positive control group animals that received only antibiotics, significant reductions in erosion depth were observed in the challenged animals administered with different concentrations of propolis, probiotics, or their combination. The highest reductions in hemorrhage, inflammation, and apoptosis were recorded in the HP + Pc group animals (challenged animals receiving 300 mg/kg of propolis and the probiotic) (P < 0.001). Overall, animals treated with lower concentrations of propolis and the mentioned probiotic mix also showed significant reductions in the evaluated histopathological parameters compared to the control group. In contrast, the least reduction in these parameters was observed in *H. pylori* challenged animals that did not receive any treatment regimen. The gastric tissues of the challenged animals that were treated with only antibiotics had higher inflammation, apoptosis, and hemorrhage scores compared to the animals that received the highest concentrations of the propolis extract combined with the probiotics (P < 0.001).

**Figure 3.**
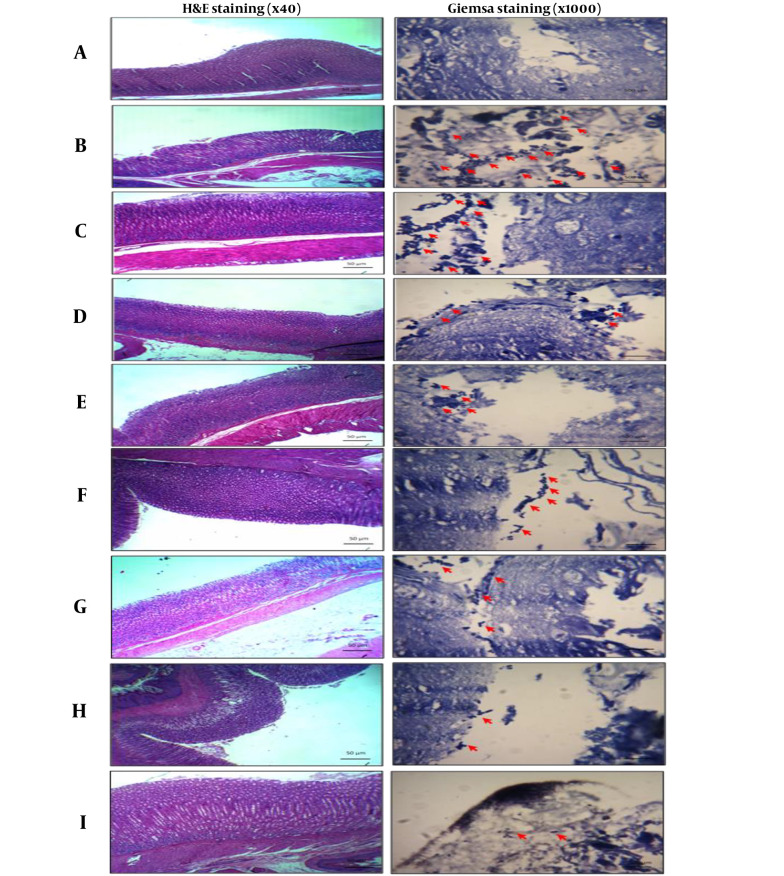
Microscopic images of *Helicobacter pylori*-induced gastropathy by H&E and Giemsa staining method 21 days after treatment of oral administrations of propolis (75, 150 and 300 mg/kg) and Probiotic (10^8^ CFU/mL) and their combination. A, control group (healthy animals); B, *H. pylori*; C, *H. pylori* + Propolis (75 mg/kg); D, *H. pylori* + propolis (150 mg/kg); E, *H. pylori* + Propolis (300 mg/kg); F, *H. pylori* + probiotic (10^8^ CFU/mL); G, *H. pylori* + propolis (150 mg/kg) and probiotic (10^8^ CFU/mL); H, *H. pylori* + propolis (300 mg/kg) and probiotic (10^8^ CFU/mL); I, positive control group (amoxicillin 50 mg/kg + clarithromycin 25 mg/kg + omeprazole 20 mg/kg). The red arrows indicate the presence of bacteria.

The microscopic images presented in Figure 3 provide visual evidence supporting the histopathological findings. According to Giemsa staining, the *H. pylori* group (B) showed more reactivity (+++), marked by the red arrow, compared to the other treatment groups. In contrast, *H. pylori* challenged animals treated with propolis and/or probiotics exhibited reduced bacterial load, as indicated by the decreased reactivity observed in the stained sections. Based on these observations, the least bacterial loads were observed in the antibiotic treatment groups, followed by the HP + Pc group animals that received the highest concentrations of propolis and probiotics. In contrast, the highest bacterial loads were observed in *H. pylori* infected rats that received no treatments, followed by challenged animals receiving either 75 or 150 mg/kg of propolis extracts, respectively (P < 0.05).

## 5. Discussion

*Helicobacter pylori *is a microorganism that thrives in acidic conditions and is linked to a range of gastric disorders. A key mechanism for its survival is the enzyme urease, which is classified as a nickel-metallo enzyme. The inhibition of urease is essential for preventing the bacterium from adhering to the gastric mucosa, particularly in the context of treating gastric ulcers (20). This study explored an alternative therapeutic strategy utilizing natural compounds such as propolis and probiotics for the treatment and eradication of *H. pylori* infection. 

Numerous natural antimicrobial agents have been the subject of extensive research, with propolis and probiotics being particularly prominent due to their antimicrobial properties (20-22). During our investigations, the examined propolis extracts demonstrated notable antibacterial activity against *H. pylori* in the agar well diffusion assay. This finding aligns with previous reports of the *in vitro* antibacterial efficacy of Brazilian propolis against *H. pylori*. Villanueva et al. assessed the inhibitory effects of 22 propolis extracts sourced from various regions in Chile against multiple strains of *H. pylori* isolated from gastric mucosa, employing both agar well diffusion and disk diffusion assays. Their findings underscored the significant antibacterial properties of the propolis extracts tested against this pathogen (23). In a similar vein, a study on Portuguese propolis from the Algarve region revealed its ability to inhibit the growth of *H. pylori* strains during the agar well diffusion assay, underscoring its potential utility in combating bacterial infections, particularly those caused by the gastric pathogen *H. pylori* (24). The antibacterial activities of propolis are dependent on the concentration of biologically active compounds, including phenolic acid esters and flavonoids such as pinocembrin and galangin (25). It is further reported that the synergistic interactions among these diverse active components play a crucial role in producing the multifaceted antimicrobial properties of propolis (26). During the study, a significant enhancement in the antibacterial activity was observed in the prepared propolis mixture (combination of all four propolis extracts). This suggests that the observed synergistic antibacterial effects are associated with the quality, quantity, and ratios of the bioactive components found in the various propolis extracts collected from different regions (27).

The probiotic bacteria are well studied for their antibacterial potential, which is related to the variety of bioactive compounds generated by these bacteria. The metabolites released in the growth medium during their growth and fermentation are commonly referred to as "postbiotic metabolites" (28). Among these metabolites, organic acids, hydrogen peroxide, and bacteriocins are believed to play a crucial role in their antibacterial activities (29). Postbiotic metabolites are recognized for their adverse impact on the morphological features of pathogenic bacteria, leading to cell death and subsequent elimination. In alignment with our findings, previous studies have documented the synergistic antibacterial effects of combined postbiotics (30, 31). 

In the present study, the supernatant fluids obtained from the mentioned probiotic strains, *L. rhamnosus* BLRH 260 and *L. reuteri* BLRE 269, showed antibacterial actions against the tested pathogen. The efficacy of these bacteria in eradicating *H. pylori* has been reported earlier (32, 33). During investigations, significant variations were observed in the degree of antibacterial effects exerted by these bacteria, as evident by measuring their inhibitory zone diameters. The notable differences observed in the inhibitory effectiveness of these probiotic strains against *H. pylori* might be linked to variations in the types and concentrations of metabolic byproducts produced by the specific probiotic bacteria (28). 

Similar to the synergistic antibacterial actions observed in the mixed propolis extracts, the antibacterial action of the probiotic mixture containing the combination of the supernatant fluids of *L. rhamnosus* and *L. reuteri* was also significantly enhanced. The enhanced activity recorded in the mixed supernatant fluids of the probiotic strains, compared to individual fractions, may be ascribed to variations in the types and concentrations of metabolic end products present in the two metabolites, which, upon combination, exhibit a synergistic effect resulting in enhanced antibacterial activity (29). 

Several studies have shown the combined effects of probiotics and propolis against a number of infectious agents. In one study, a group of researchers investigated the antimicrobial efficacy of probiotics in conjunction with propolis as potential treatments against *Enterococcus faecalis* (34). Their findings indicated that the proliferation of *E. faecalis* was markedly reduced in all treated groups compared to the untreated control, with the combination of probiotics and propolis demonstrating the most significant reduction in pathogen growth. Based on this research and the results of our in vitro antibacterial analysis that showed both propolis and probiotics to be highly effective in eliminating *H. pylori*, we aimed to investigate the combined effects of probiotics and propolis on *H. pylori* in challenged Wistar rats. The results obtained from this study provided important insights into the effects of propolis and probiotic treatments on body weight and histopathological changes in *H. pylori*-induced gastropathy.

During preliminary in vivo analysis, the body weight of animals in various treatment groups was assessed to evaluate the differences in the efficacy of the treatments administered. A significant enhancement in the weight of animals receiving a combination of probiotics and propolis extracts was observed. Furthermore, the impact of probiotics on weight gain was found to be more substantial compared to that of propolis. Contradictory results have been reported for the effects of propolis on body weight in animals. Studies conducted by Waly et al. and Sierra-Galicia et al. demonstrated that propolis administration increased body weight in rabbits (35, 36), while similar observations were recorded in propolis-fed lambs (37). In contrast, Daneshmand et al. reported that the addition of propolis extract to the diet led to a reduction in the body weight of broiler chickens (38). In alignment with these results, we noted a decrease in the weight of rats administered propolis compared to those receiving either probiotics or a combination of propolis and probiotics. Furthermore, during the course of the study, a time-dependent effect of the treatments was observed in all treatment groups, which might be due to the fact that, aside from the effects of *H. pylori* infection, stress parameters could have been imposed on animals that caused loss of appetite and reduction in weight. Nevertheless, with the passage of time, recovery was observed, accompanied by an increase in the appetite of the animals, leading to weight gain. Notably, these findings indicate the efficacy of the treatment regimen and the dosage in the treatment of *H. pylori*.

During histopathological studies, further insights into the extent of damage caused by *H. pylori* infection and the effectiveness of different treatments were revealed. The animals treated with either propolis, probiotics, or their combination had significantly lower erosion, hemorrhage, inflammation, and apoptosis scores compared to the untreated *H. pylori*-challenged rats. Both propolis and probiotics have demonstrated the ability to reduce the virulence factors associated with *H. pylori* and decrease the production of pro-inflammatory cytokines in infected murine models, thereby alleviating gastric damage (5). Song et al. indicated that treatment with propolis led to a reduction in inflammation and gastric epithelial injury in a mouse model of *H. pylori*-induced gastritis (4). Meanwhile, Bai et al. reported that probiotic supplementation lessened gastric inflammation and enhanced gastric histology in mice infected with *H. pylori* (39). Notably, in contrast to the antibiotic treatment group animals, both propolis and probiotics showed significantly lower severity of erosion, hemorrhage, inflammation, and apoptosis resulting from *H. pylori* infection.

In the gastric tissue samples of the animals treated with propolis and/or probiotics, reduced bacterial load comparable to the antibiotic-treated group animals was observed. Boyanova et al. showed that propolis extract exhibits strong inhibitory effects on *H. pylori* growth, comparable to antibiotics (17). According to Romero et al., polyphenol compounds of propolis have significant enhancing effects on the eradication rate of *H. pylori* in rats with gastritis and peptic ulcers, suggesting that propolis supplementation may enhance the effectiveness of conventional *H. pylori* treatment and that polyphenol compounds act as the main compounds (16). The antibacterial effects of probiotics against *H. pylori* and their role in mitigating the side effects associated with antibiotic use have also been reported (15, 16, 40, 41).

In summary, the combination of probiotics with the highest dosage of propolis (300 mg/kg) exhibited the most substantial effect in diminishing *H. pylori* infection in rats. This synergistic interaction is likely attributed to the complementary mechanisms of action of both propolis and probiotics. Propolis contains caffeic acid phenethyl ester (CAPE), which inhibits the enzyme peptide deformylase, a critical factor for the survival of *H. pylori* (42). Conversely, the underlying mechanism for probiotics that might contribute to their positive impacts on *H. pylori* eradication includes their ability to bolster immune responses, restore gut microbiota, and generate lactic acid (43, 44). A dosage-dependent effect of propolis was noted, with the highest concentration demonstrating the most significant impact in alleviating the histopathological changes induced by *H. pylori* infection. The dosage-dependent effect of propolis has been observed in other studies as well (45, 46).

### 5.1. Conclusions

The results of the study elucidate the synergistic effects of orally administered propolis and probiotics on body weight and improvements in the histopathological changes associated with *H. pylori* infection. It is crucial to recognize that variations in dosage, treatment duration, animal models, and experimental design may affect the interpretation and comparability of results across different studies. Therefore, further research is essential to determine the underlying mechanisms and optimize dosage and treatment protocols for maximum efficacy. These findings could pave the way for the development of beneficial food products utilizing probiotics, postbiotics, and propolis to address digestive issues caused by pathogenic bacteria in the gastrointestinal system.

## Data Availability

The dataset presented in the study is available on request from the corresponding author during submission or after its publication.
